# Selective inhibition of NADPH oxidase reverses the over contraction of diabetic rat aorta^☆^

**DOI:** 10.1016/j.redox.2013.12.002

**Published:** 2013-12-11

**Authors:** Atif ur Rehman, Elma Dugic, Chris Benham, Lisa Lione, Louise S. Mackenzie

**Affiliations:** a Biosciences, School of Life and Medical Sciences, University Hertfordshire, College Lane AL10 9AB, UK; b Department of Medical and Health Sciences, Faculty of Health Sciences, Linköping University, 58185 Linköping, Sweden

**Keywords:** Reactive oxygen species (ROS), NADPH oxidase (NOX), Apocynin, VAS2870, Aorta, Streptozotocin (STZ)

## Abstract

Abnormal vascular responsiveness in diabetes has been attributed to a number of changes in contractile pathways, affected in part by the overproduction of reactive oxygen species (ROS). It has been reported that NADPH oxidase (NOX) is increased in diabetic (streptozotocin treated; STZ) rat arteries; however the pharmacological agents used to inhibit NOX activity are known to be unsuitable for in vitro studies and have a high level of non-selectivity. Here we have used the highly selective NOX inhibitor VAS2870 in diabetic rat aorta and compared its effects with apocynin, SOD, and allopurinol on phenylephrine and U46619 induced contraction.

Male Wistar rats were injected intraperitoneally with 65 mg/kg STZ and development of diabetes was confirmed by testing blood glucose levels. Rats were killed by CO_2_ asphyxiation, and the thoracic aorta removed and mounted in an organ bath under a tension of 1 g. Diabetic rat aortas exhibit a greatly increased response to phenylephrine, which was reduced to a level consistent with control rat aorta by 10^−5^ M VAS2870 and 150 U/ml SOD. Incubation with VAS2870 led to an increase in normal rat aorta contraction, but led to a significant reduction in phenylephrine and U46619 induced tone in diabetic rat aorta, which indicates that ROS in diabetic rats directly contributes to these contractile responses. Apocynin and allopurinol had no effect on contraction in diabetic or normal rat aorta. This data is the first to show that selective inhibition of NOX reduces diabetic arterial contraction in direct comparison with inhibition of other known contributors of ROS.

## Highlights

• An increase in ROS production in arteries from diabetic rats has been attributed to NOX, although the pharmacological tools to show this are poor.
• VAS2870 is a relatively new NOX inhibitor that has better selectivity for NOX than apocynin, and can be used in vitro systems.
• VAS2870 significantly reduced the over contraction of diabetic rat aorta induced by phenylephrine and U46619 to levels consistent with aorta from normal rats.
• Apocynin had no effect on the aorta contractions.

## Graphical abstract

**Figure:**
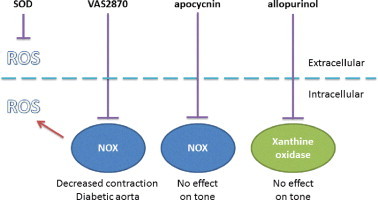

## Introduction

The study of reactive oxygen species (ROS) in vascular biology has been hampered in part by the lack of availability of selective NADPH oxidase (NOX) inhibitors. Apocynin (1-(4-hydroxy-3-methoxyphenyl)ethanone) is a widely used NOX inhibitor which acts by blocking NOX assembly and thus needs to be administered orally to have a full effect. There are a range of non-selective modes of action attributed to apocynin, including inhibition of RhoA [13] and direct action as an antioxidant in vascular smooth muscle cells and endothelial cells [7], which taken together has called into question the use of this inhibitor [7,13]. New molecules have been developed, such as the cell-permeable pan NOX inhibitor VAS2870 (7-(2-benzoxazolylthio)-3-(phenylmethyl)-3H-1,2,3-triazolo[4,5-d]pyrimidine), which abolishes ROS production without altering baseline ROS [14].

Vascular damage induced by diabetes has been attributed to the increased production of ROS by NOX [11,17], however the pharmacological evidence relies on NOX inhibition with apocynin. In this study, we used inhibitors of ROS production, VAS2870, apocynin, superoxide dismutase (SOD) and the xanthine oxidase inhibitor allopurinol on phenylephrine and U46619 induced contraction in diabetic and control rats.

## Materials and methods

The care and use of all rats in this study was carried out in accordance with UK Home Office regulations, UK Animals (Scientific Procedures) Act of 1986 under PPL70/6788. Male Wistar rats (350–450 g) were given a single injection of 65 mg/kg intraperitoneally (i.p., DV 10 ml/kg). Streptozotocin (STZ) dissolved in 20 mM citrate buffer (pH 4.5). For 48 h following STZ or control injection, additional 2% sucrose solution was provided, to avoid the initial hypoglycaemia that is seen following STZ. Rats were monitored for a minimum of 2 weeks post-injection, and diabetes was confirmed by testing a drop of tail vein blood; and rats showing an elevated blood glucose of >16 mmol/L were considered diabetic.

The rats were killed by CO_2_ asphyxiation and their thoracic aortas were rapidly removed and dissected in Kreb's buffer (pH 7.4, NaCl 118 mM, KCl 4.7 mM, MgSO_4_ 1.2 mM, KH_2_PO_4_ 1.2 mM, CaCl_2_ 2.5 mM, NaHCO_3_ 25.0 mM and glucose 11.0 mM). Aortas were cut into 2 mm-wide rings after removing the surrounding connective tissue and fat. Each ring was suspended with silk thread in a 20 ml organ bath filled with Kreb's buffer and maintained at 37 °C. The upper end of the wire was connected to a force displacement transducer and the lower one connected to an L-shaped mounting hook. The bath solution was continuously bubbled with 95% O_2_ and 5% CO_2_. An equilibration time of 1 h was employed during which the aorta rings were tensioned to 1 g.

Arteries were incubated with 10^−5^ M 3-benzyl-7-(2-benzoxazolyl)thio-1,2,3-triazolo(4,5-d)pyrimidine (VAS-2870), 10^−5^ M apocynin, 150 U/ml SOD, 10^−5^ M allopurinol or vehicle control for 30 min, followed by increasing concentrations of phenylephrine (10^−8^ to 10^−5^ M) and U46619 (10^−9^ to 3×10^−7^ M).

### Statistical analysis

All data are expressed as mean±standard error of the mean (SEM). Data was analysed by two-way ANOVA for repeated measurements followed by Bonferroni's post-hoc test, using GraphPad prism version 4. Differences were considered to be statistically significant when *p*=<0.05.

## Results

**Fig. 1:**
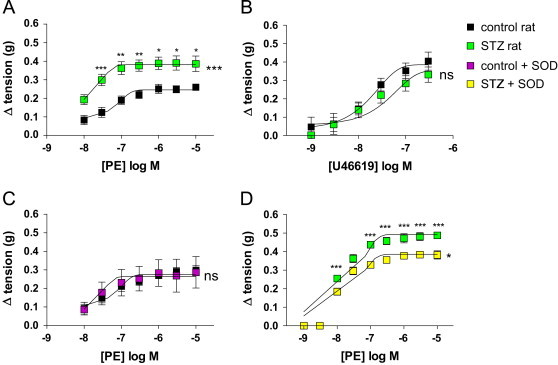
The effects of phenylephrine and U46619 on aorta from diabetic and normal rats. Change in tension (in g) induced by (A) phenylephrine and (B) U46619 in control and diabetic rat aorta; *n*=12. Effects of incubation with 150 U/ml SOD on phenylephrine induced contraction in aorta from (C) normal rat aorta and (D) diabetic rat *n*=4. Data are presented as mean±SEM. Significance by two-way ANOVA and Bonferroni's post hoc test is denoted by ^⁎^=*p*<0.05, ^⁎⁎^=*p*<0.01, ^⁎⁎⁎^=*p*<0.001 ns, not significant; compared to respective group within figure.

At the time of the experiment, rat biometrics were measured, and the average weight of rat had significantly reduced from a mean of 455 g±15 in control rats compared to 383 g±11 (*n*=12; ^**^p<0.01 by *T*-test). Blood glucose measurements increased from 6.68 mM±0.28 in control rats to 26.61 mM±1.35 (*n*=12; ^***^p=0.001) in diabetic rats. Phenylephrine induced contraction of aorta from diabetic treated rats is significantly increased compared to vehicle treated control rats (Fig. 1A), in comparison to U46619 induced contraction which shows no difference between diabetic and control rat aorta (Fig. 1B). To ascertain whether the increase in contraction in diabetic rat aorta was due to ROS production, aorta were incubated with 150 U/ml SOD, which did not affect contraction in normal rat aorta (Fig. 1C), but significantly reduced phenylephrine induced in diabetic rat aorta (Fig. 1D). SOD had no effect on U46619 induced contraction from either type of rat (data not shown).

**Fig. 2:**
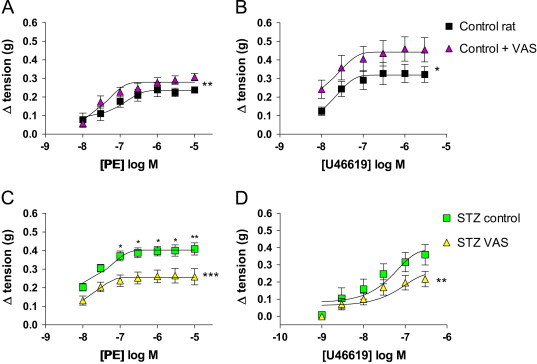
Contractile responses in aorta incubated with 10^−5^ M VAS2870. Change in tension (in g) induced by (A) phenylephrine and (B) U46619 in normal rat aorta following incubation with 10^−5^ M VAS2870; change in tension following VAS2870 on (C) phenylephrine and (D) U46619 in aorta from diabetic rats. Data are presented as mean±SEM; *n*=6–7 for all groups. Significance by two-way ANOVA and Bonferroni's post hoc test is denoted by ^⁎^=*p*<0.05, ^⁎⁎^=*p*<0.01, ^⁎⁎⁎^=*p*<0.001 ns, not significant; compared to respective group within figure.

There were no changes in U46619 or phenylephrine induced contraction in normal or diabetic rat aorta following incubation with 10^−5^ M apocynin or 10^−5^ M allopurinol (data not shown). In contrast, incubation with 10^−5^ M VAS2870 resulted in an increase in phenylephrine (Fig. 2A) and U46619 (Fig. 2B) induced contraction in normal rat aorta. In contrast, VAS2870 significantly reduced phenylephrine (Fig. 2C) and U46619 (Fig. 2D) responses in diabetic aorta.

## Conclusion

This study demonstrates that selective inhibition of NOX in the STZ model of diabetes reduces contraction by phenylephrine to a level consistent with control aortas. It has been well established in the literature that diabetes leads to wide spread vascular damage, attributed largely to the overproduction of ROS in the vasculature [5], which in turn has been attributed to the up-regulation of NOX [8]. NOX has become a therapeutic target [4], yet up until recently, one of the most widely used NOX inhibitors apocynin, which inhibits the assembly of NOX, has been used in in vitro pharmacological studies, even though its effects can no longer be attributed to NOX inhibition. Indeed, apocynin has been shown to act directly as an antioxidant in vascular tissues [7], and in our hands apocynin had no effect on aorta contraction to U46619 or phenylephrine. In order to study the effects of NOX inhibition, we chose the diabetic STZ rat aorta as a model of vascular damage induced by overproduction of ROS.

ROS has been shown to contribute to α-adrenergic contractile responses in endothelium denuded rat tail artery [15]. The α adrenergic agonist norepinephrine elicits a significantly increased contraction in diabetic rat aorta and mesenteric arteries [10], which taken together fits well with our data presented here that ROS contributes to phenylephrine induced contraction of diabetic and not normal rat aorta. The diabetic rat aorta exhibited a significantly increased response to phenylephrine compared to control aorta, in keeping with previous similar findings [16]. Interestingly SOD significantly reduced phenylephrine responses in diabetic and not control rat aorta, indicating that the levels of ROS released under control conditions is minimal, but significantly increased in diabetic rats. This supports recent work showing that deleterious ROS production is extracellular [6]. However, our findings contrast with Anozie et al., who showed that SOD significantly decreased phenylephrine contraction from diabetic rat aorta [1], although this may be explained by the authors using double the units of SOD than in the study presented here.

There has been some discussion in the literature as to whether xanthine oxidase contributes to ROS production in the diabetic rat [12] or is not involved [3]. While a recent study shows that xanthine oxidase is up regulated in diabetic rat myocardium [9], our data indicates that xanthine oxidase inhibition with allopurinol has no effect on U46619 or phenylephrine mediated contraction.

Inhibition of ROS with the inhibitors VAS2870, apocynin, allopurinol and SOD had no further effect on U46619, indicating that ROS is not involved in TP receptor contraction in normal or diabetic rat aorta. In our study, we found that rat aorta responses to U46619 remains unchanged following the development of diabetes, which suggests that TP receptor signalling is not affected by ROS production. This contrasts with previous findings where it was shown that U46619 induced contraction of STZ rat aorta is significantly increased [2].

Of central interest to this data is the use of VAS2870 to inhibit NOX production of ROS in the aorta. Surprisingly, the use of VAS2870 significantly increased the phenylephrine and U46619 mediated responses in control rat aorta. It is well known that the main dilatory hormone in aorta is nitric oxide, whose biological activity is critically influenced by the production of ROS and the quenching effects of superoxide radicals with nitric oxide results in the formation of peroxynitrite. In control tissues therefore, VAS2870 inhibited NOX and the production of ROS inside the cell, where normally it would react with basally produced nitric oxide; the reduction in ROS production lead to an increase in contraction most likely due to the loss of ROS and peroxynitrite production, and the removal of basally produced nitric oxide.

In contrast, the diabetic rat aorta contraction to phenylephrine and U46619 was significantly reduced by the presence of VAS2870, to a level consistent to a control rat level, which indicates that ROS in diabetic rats contributes to these contractile responses directly. This data is the first to show that selective inhibition of NOX reduces diabetic arterial contraction in direct comparison with inhibition of other known contributors of ROS, and supports the use of selective NOX inhibitors in the development of new therapies for diabetes.
